# Out-diffused silver island films for surface-enhanced Raman scattering protected with TiO_2_ films using atomic layer deposition

**DOI:** 10.1186/1556-276X-9-398

**Published:** 2014-08-15

**Authors:** Semen Chervinskii, Antti Matikainen, Alexey Dergachev, Andrey A Lipovskii, Seppo Honkanen

**Affiliations:** 1 Institute of Photonics, University of Eastern Finland, P.O. Box 111, Joensuu FI-80101, Finland; 2 Institute of Physics, Nanotechnology and Telecommunications, St. Petersburg State Polytechnic University, 29 Polytechnicheskaya, St. Petersburg 195251, Russia; 3 Ioffe Physical-Technical Institute of the RAS, 26 Polytekhnicheskaya, St. Petersburg 194021, Russia; 4 Department of Physics and Technology of Nanostructures, St. Petersburg Academic University, 8/3 Khlopina, St. Petersburg 194021, Russia

**Keywords:** Surface plasmon resonance, Nanoparticle, Nanoisland, Film, Self-assembly, Dielectric spacer

## Abstract

We fabricated self-assembled silver nanoisland films using a recently developed technique based on out-diffusion of silver from an ion-exchanged glass substrate in reducing atmosphere. We demonstrate that the position of the surface plasmon resonance of the films depends on the conditions of the film growth. The resonance can be gradually shifted up to 100 nm towards longer wavelengths by using atomic layer deposition of titania, from 3 to 100 nm in thickness, upon the film. Examination of the nanoisland films in surface-enhanced Raman spectrometry showed that, in spite of a drop of the surface-enhanced Raman spectroscopy (SERS) signal after the titania spacer deposition, the Raman signal can be observed with spacers up to 7 nm in thickness. Denser nanoisland films show slower decay of the SERS signal with the increase in spacer thickness.

**PACS:**

78.67.Sc (nanoaggregates; nanocomposites); 81.16.Dn (self-assembly); 74.25.nd (Raman and optical spectroscopy)

## Background

Metal island films (MIFs) have attracted significant attention due to the strong surface plasmon resonance (SPR) effect in these nanoislands. The spectral position of the SPR is influenced and can be tuned by the MIF density as well as the substrate and cover materials used [1-3]. Surface-enhanced Raman spectroscopy (SERS) in biological and chemical sensing [4] can be regarded as one of the most intriguing applications of MIFs. It can provide at least 10^10^- to 10^12^-fold intensity enhancement compared to the normal Raman scattering [3]. The main reason for this intensity enhancement is the electromagnetic (EM) enhancement mechanism prevailing over the chemical enhancement (CHEM) by several orders of magnitude [3]. This is because the EM enhancement is proportional to about the forth power of the SPR-increased local electric field input in Raman scattering, i.e., in the analyzed media adsorbed on the MIF (an adsorbate), while the reported CHEM enhancement factors, due to metal island-adsorbate interaction, are approximately 10^2^. It is essential to decrease the distance between separate metal islands in a MIF, which results in the increase of the local electric field intensity and, consequently, in a larger SERS signal [5]. Other prospective applications of MIFs include catalysis [6,7], photovoltaics [8], and fluorescence enhancement [9]. For many practical uses, MIFs should be protected with a dielectric cover, which influences not only the CHEM but also the EM enhancement of SERS through the change of local electric field in adsorbates. At the same time, cover-induced shifts of the SPR spectral position can be used to tune SERS measurements for a specific wavelength, which is of high importance for surface-enhanced resonance Raman scattering [10]. The influence of MIF dielectric covers (spacers between the MIF and an analyte) on SERS intensity has been studied for more than two decades [11]. However, only the recent use of a very precise atomic layer deposition (ALD) technique has allowed obtaining quantitative results related to the SERS influence by alumina spacers deposited on metal microspheres [3], MIFs [12], and metal nanowires [13]. However, due to the difference in metal nanoislands and nanoparticles used in the experiment, these results can hardly be compared, and they contradict the data obtained in SERS experiments using MIFs covered with non-ALD spacers [14]. It is worth to note that the key issues in this comparison are the SPR shift and the local electric field decay vs the spacer thickness. It is worth to note that dielectric-capped isolated metal nanospheres have already demonstrated their effective applicability in photovoltaics [15] and SERS [16].

Here we present our studies on the influence of a high-index TiO_2_ ALD spacer on the SPR position and SERS intensity in the case of silver island films grown on soda-lime glass substrates using our recently developed silver out-diffusion (SOD) technique [17]. It is important to note that MIFs are highly fragile and, therefore, they must be protected for any practical use. The use of conformally grown ALD films is ideal for protecting MIFs with a cover layer, since the layer thickness can be controlled at an atomic level and the initial surface relief structure can be maintained with thin cover layer thicknesses [18]. In the experiments, we varied the thickness of the ALD TiO_2_ spacer and the MIF structure. The interest in TiO_2_ spacers is twofold: (1) the high catalytic abilities of TiO_2_[19-21] allowing the use of SERS with a titanium dioxide spacer in nanoscale organic and biochemistry studies and (2) the high refractive index of TiO_2_ providing stronger control of the ALD-coated MIF structure, which results in wider spectral tunability of the system.

## Methods

### MIF formation and characterization

We fabricated silver nanoisland films using SOD from glass in the course of the ion-exchanged glass substrate annealing in a reducing hydrogen atmosphere. In the experiments, we used soda-lime glass microscope slides produced by Menzel [22]. The silver-sodium ion exchange was performed at 325°C in an ion-exchange bath containing 5 wt.% of silver nitrate and 95 wt.% of sodium nitrate as was reported elsewhere [23]. One-millimeter-thick slides with a size of 20 × 30 mm^2^ were immersed in the melt for 20 min, which provided a few microns of silver penetration depth in the glass. Optical absorption spectroscopy of the ion-exchanged slides did not show any absorption peaks in the spectral range corresponding to the surface plasmon resonance, which indicated the absence of silver nanoparticles both in the bulk and on the surface of the slides. The ion-exchanged slides were annealed in hydrogen for 10 min to reduce silver ions to atoms and get a supersaturated solid solution of neutral silver in the glass matrix. According to the proposed mechanism [24], this results in the formation of both silver nanoparticles within the glass and a silver island film on the glass surface (MIF) due to the out-diffusion of silver atoms.

After the MIF formation, we measured the optical absorption spectra of the samples using a Specord 50 spectrophotometer (Analytik Jena AG, Jena, Germany). To distinguish the MIF optical absorption and absorption of light by silver nanoparticles formed in the bulk of the glass slides, we subtracted the spectra of the samples after the surface film removal from the spectra measured after the processing of the glass slides in hydrogen atmosphere. The fragility of the MIFs allowed cleaning the glass surface from the nanoislands using just cotton with acetone. The topography of the MIFs was characterized with a Veeco Dimension 3100 atomic force microscope (AFM; Veeco Instruments Inc., Plainview, NY, USA), which allowed studying both the shape of separate silver islands and their size and distribution corresponding to different SOD regimes.

### Atomic layer deposition and characterization

ALD was used to coat the MIF samples with thin layers of titanium dioxide. TiO_2_ was chosen for its high refractive index (*n* = 2.27) strongly influencing the SPR wavelength and because of its applicability for photocatalysis. Films were deposited at 120°C with Beneq TFS-200 reactor (Beneq, Espoo, Finland) using titanium tetrachloride (TiCl_4_) and water (H_2_O) as precursors, and between each deposition cycle, a nitrogen purge was used to remove extra precursor materials from the reactor chamber.

The samples covered with TiO_2_ film of different thicknesses were also characterized with a Specord 50 spectrophotometer and a Veeco Dimension 3100 atomic force microscope.

### Surface-enhanced Raman scattering measurements

Signal enhancement properties of the MIF samples were examined using rhodamine 6G as a target molecule. Five-microliter droplets of 1 μM rhodamine (diluted in water) were deposited on all samples and allowed to dry forming an analyte-covered circular area of 4 to 5 mm in diameter. Raman scattering was measured using an inVia Raman microscope system (Renishaw, Gloucestershire, UK) with a 514-nm excitation laser. The beam was focused into an approximately 5-μm spot, and for each sample, nine measurements were performed from an area of 50 × 50 μm^2^ and the spectra were collected using an optical power of 50 μW and exposure times of 10 and 20 s for the uncoated and coated samples, respectively. The collected spectra were averaged and the background fluorescence was subtracted using an asymmetric least squares smoothing.

## Results and discussion

### Structure and optical absorption of initial MIF

**Figure 1:**
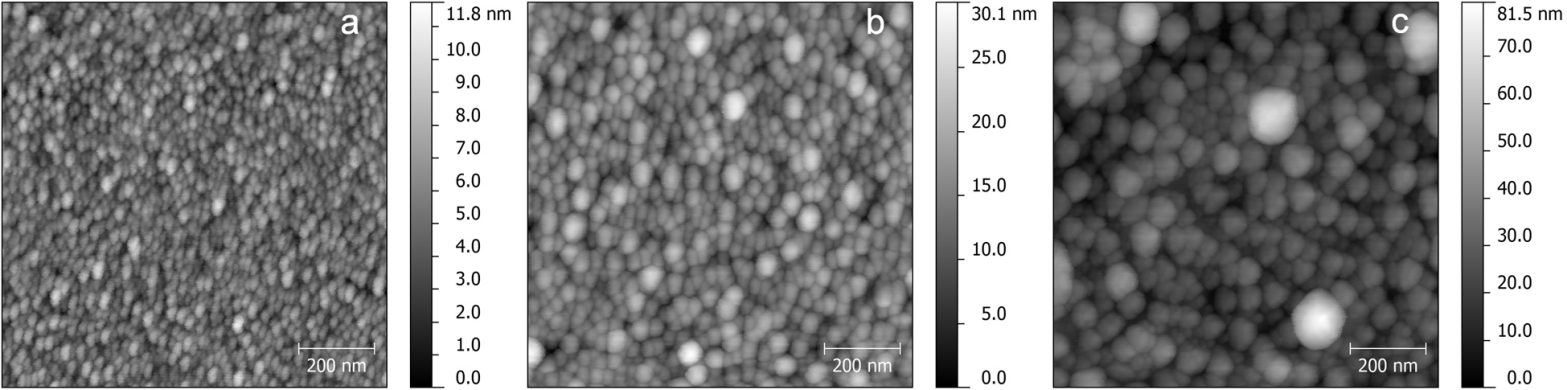
AFM images of MIFs prepared using annealing in hydrogen at 150°C (a), 250°C (b), and 300°C (c).

AFM studies of SOD MIF samples allowed concluding that depending on the mode of SOD we can fabricate MIFs consisting of tiny (approximately 10 nm), nearly isolated silver nanoislands (Figure 1a), bigger islands which can be placed very closely (Figure 1b), and partly coagulated nanoislands (Figure 1c).

**Figure 2:**
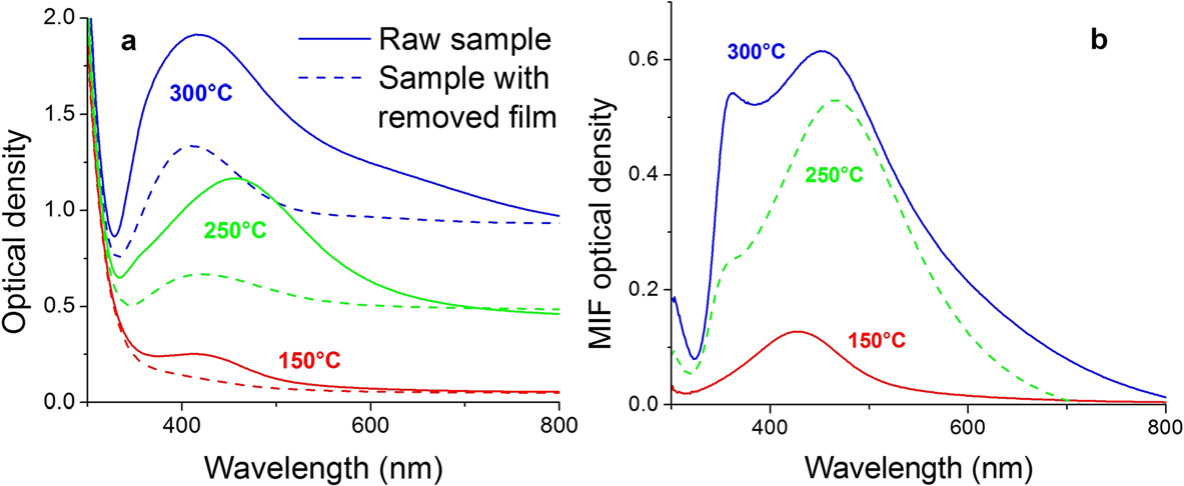
**Optical absorption spectra and differential spectra.** Optical absorption spectra of samples with a MIF prepared using annealing in hydrogen at 150°C, 250°C, and 300°C before (solid line) and after (dashed line) the MIF removal **(a)** and the differential spectra corresponding to the MIFs themselves **(b)**.

The optical absorption spectra of the prepared samples and the spectra of MIFs obtained using subtraction of spectra measured with and without the MIF are presented in Figure 2. One can see that the shape and position of the SPR peak in the absorption spectra are strongly influenced by the processing mode, but generally higher temperature of SOD results in higher SPR absorption. The SPR peak moves from 420- to 470-nm wavelength with the increase of processing temperature from 150°C to 250°C. Further increase in SOD temperature does not move the peak so much. The position of the SPR peak corresponds to the size of silver islands, the bigger is the size the longer is the SPR wavelength [17]. The peculiarities of the spectra in the 350- to 370-nm region can be attributed to the quadrupole plasmon resonance [24], the absorption of atomic silver, and the proximity of this region to the absorption edge of silver ion-enriched glass. The latter may result in artifacts in the differential spectra. It should be noted that no peculiarities in the 350- to 370-nm range were observed in raw spectra measured after SOD.

### Optical absorption and structure of MIF with TiO_2_ cover

**Figure 3:**
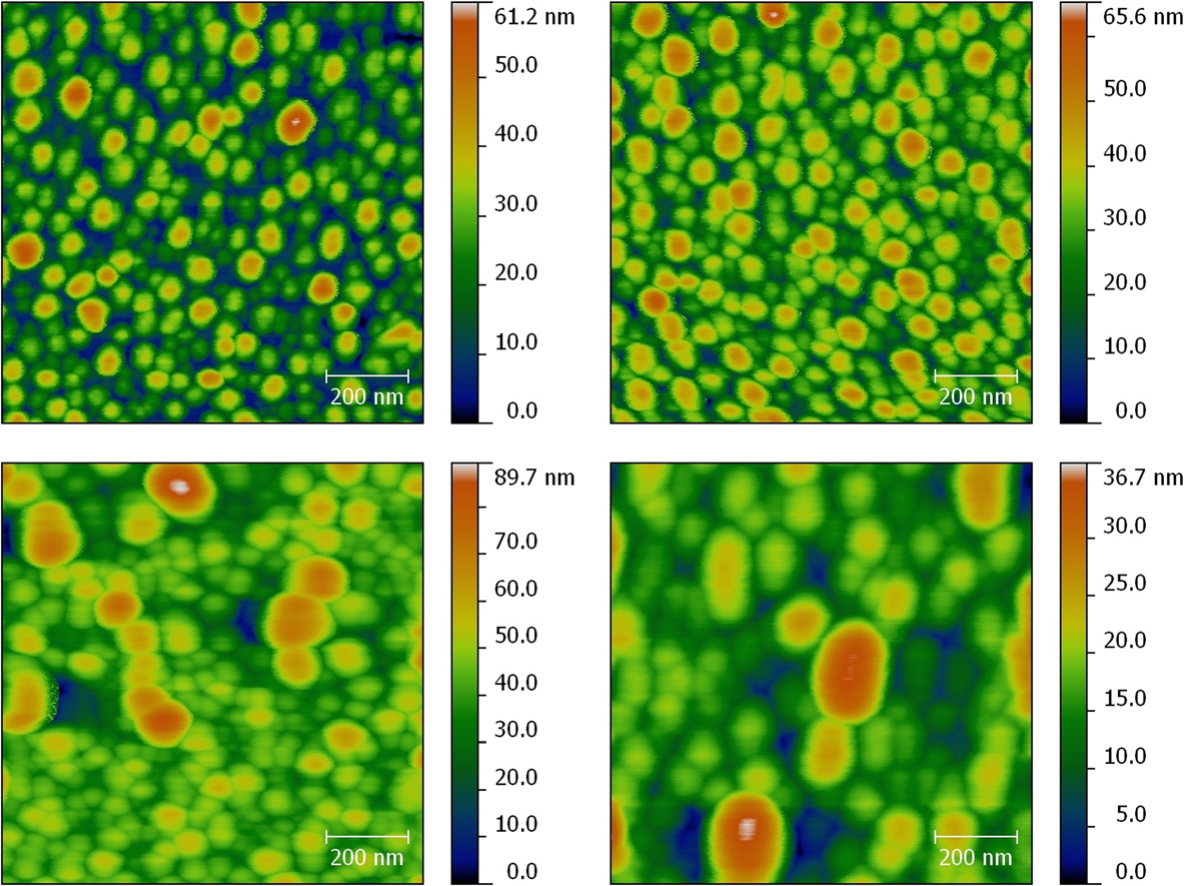
**AFM images of MIFs.** The MIFs were prepared using annealing in hydrogen at 250°C and coated with 3-nm (top left), 10-nm (top right), 50-nm (bottom left), and 200-nm (bottom right) TiO_2_.

AFM characterizations performed after TiO_2_ deposition (see Figure 3) revealed that the surface profile formed by silver nanoislands becomes smoother very slowly with the increase in the thickness of the ALD layer. The relief of the ALD-covered MIF is very close to the relief of the initial MIF for thinner films, and it stays unsmooth and critically related to the relief of the MIF even up to 200-nm ALD film thicknesses. This behavior was the same for all studied MIFs.

**Figure 4:**
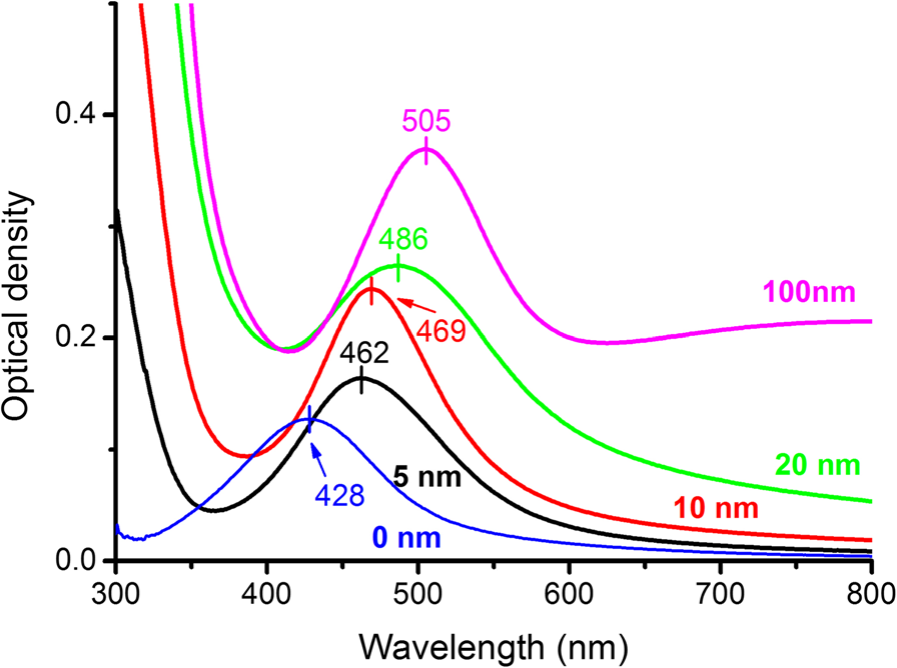
**Optical absorption spectra of the films.** The films were prepared using annealing in hydrogen at 150°C and coated with ALD-TiO_2_ of different thicknesses as marked near the curves. The substrate spectrum is subtracted. The SPR positions are indicated with the lines.

The optical absorption spectra of the TiO_2_-covered MIFs demonstrate the shift of the SPR peak towards a longer wavelength, as illustrated in Figure 4.

**Figure 5:**
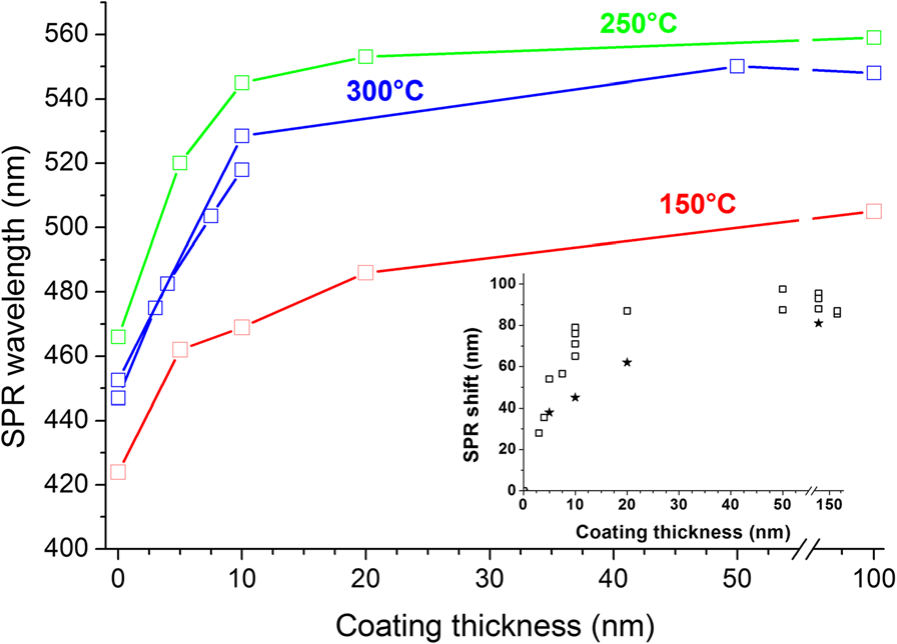
**The position of surface plasmon resonance vs the thickness of TiO**_**2 **_**cover.** For MIFs prepared using annealing in hydrogen at 150°C, 250°C, and 300°C. The absorption spectra of initial MIFs are presented in Figure 2b. Inset: the SPR shift vs the cover thickness for all prepared samples; stars denote the samples annealed at 150°C, the smallest silver islands.

In Figure 5, the SPR wavelength found using the spectra decomposition is plotted as a function of the ALD TiO_2_ cover thickness. One can see that the shift of the SPR saturates for thicker films; however, it is difficult to conclude about the exact thickness corresponding to the saturation. Nevertheless, this thickness exceeds approximately 40 nm, and the shift is bigger for the MIFs with the SPR position at longer wavelengths (see the inset in Figure 5).

### SERS studies: TiO_2_ spacer and MIF structure influence

**Figure 6:**
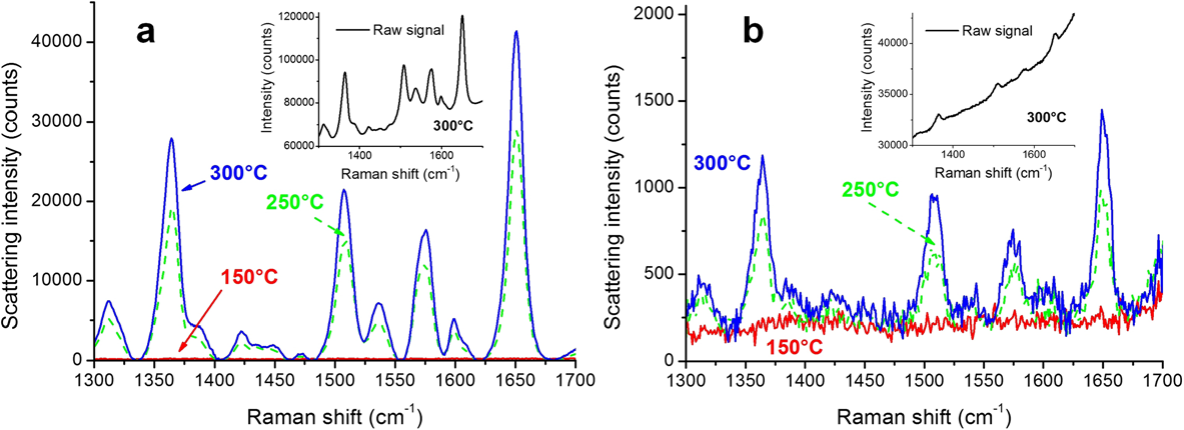
**SERS spectra of rhodamine 6G.** Rhodamine 6G was deposited onto uncoated **(a)** and coated with 3-nm TiO_2_**(b)** films prepared using annealing in hydrogen at 150°C, 250°C, and 300°C. Measurement power 50 μW, spot diameter 5 μm, and exposure time 10 s. Insets: raw signal with background fluorescence.

**Figure 7:**
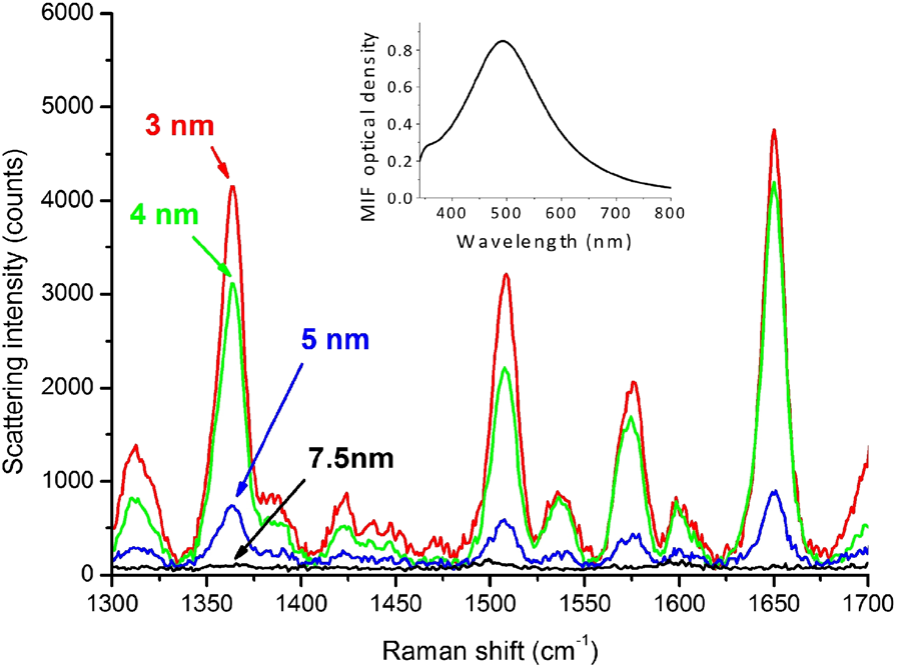
**SERS spectra of rhodamine 6G.** Measured using the TiO_2_-covered sample prepared using annealing in hydrogen at 250°C for different spacer thicknesses. Measurement power 50 μW, exposure time 20 s, and approximate spot size 5 μm. Inset: absorption spectrum of the initial MIF.

**Figure 8:**
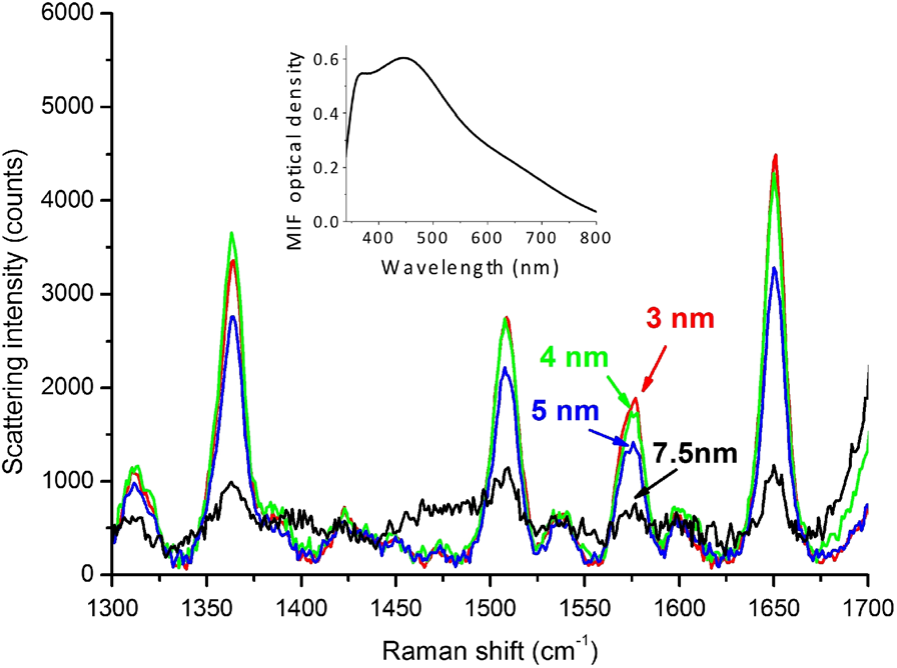
**SERS spectra of rhodamine 6G.** Measured using the TiO_2_-covered sample prepared using annealing in hydrogen at 300°C for different spacer thicknesses. Measurement power 50 μW, exposure time 20 s, and approximate spot size 5 μm. Inset: absorption spectrum of the initial MIF.

SERS studies of the rhodamine 6G deposited on initial and covered MIFs showed that after the ALD of a 3-nm TiO_2_ spacer the measured SERS signal dropped about 1.5 orders of magnitude (samples annealed in hydrogen at 150°C, 250°C, and 300°C), as is illustrated in Figure 6. The spacer influence on the SERS intensity is illustrated in Figure 7.To compare the spacer effect on the SERS signal obtained using differing MIFs, we performed similar measurements using a denser MIF (sample annealed in hydrogen at 300°C). The results of these measurements are presented in Figure 8. Comparing Figures 7 and 8, one can see that the influence of the spacer thickness is weaker in the case of a denser MIF, that is, the SERS signals go down slower.

## Discussion

**Figure 9:**
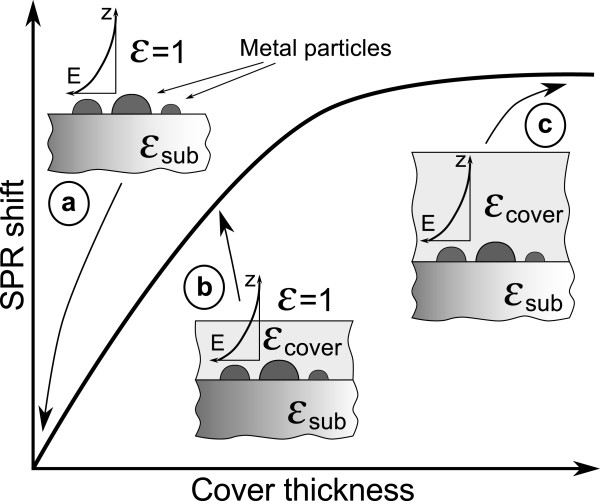
Schematic of SPR electric field localization (lateral component) in MIF for different dielectric cover thicknesses.

The MIF formation occurs because the glass surface is a stronger sink for neutral silver atoms than the arising nuclei of metal silver in the bulk of the glass [25]. Thus, lowering the temperature and shortening the duration of hydrogen processing can provide prevailing of the MIF over the nanoparticles in the bulk of the glass growth. Varying the hydrogen annealing temperature and duration allowed us to grow MIFs differing in silver nanoisland size and concentration. It is worth to note that longer SOD duration results in simultaneous increase of concentration and size of silver nanoislands. The position of SPR in the SOD-made MIFs falls in the spectral range below 500 nm, the exact position of the SPR being dependent on the mode of the MIF preparation. These MIFs demonstrate their applicability in SERS and being covered with up to 7.5-nm-thick titania layers allow registering below a monolayer of rhodamine 6G. After ALD of titania, the shift of the SPR occurs in the TiO_2_-covered MIFs. This is due to the change in the dielectric surrounding of silver nanoislands. In our case, their shape is very close to a hemispherical one [17] and the shift occurs in the same way as in the case of spherical nanoparticles [26]. The origin of this shift is the loading of the electron-electric field oscillating system with a higher permittivity dielectric. Since the index and permittivity of the titanium dioxide film exceed the ones of air, the SPR frequency drops and, respectively, the resonant wavelength increases. This situation is seen particularly clearly with thicker TiO_2_ layers. To evaluate this spectral shift, one should solve the electromagnetic problem describing the geometry presented in insets a-c in Figure 9. However, there still is no any exact solution for this problem, and the reported numerical calculations [27] performed for an isolated hemisphere in a uniform dielectric surrounding (*ϵ*_sub_ = *ϵ*_cover_) have shown that even in this case about 1% rounding of the hemisphere edge results in a meaningful shift of the resonant frequency. In measurements, it is difficult to characterize the curvature of the edges of a nanoisland formed in SOD on a glass substrate, and this does not allow constructing a numerical model for this situation. We can only assume that the shapes of the nanoislands in differently prepared MIFs are very similar. This is indeed indicated by the inset in Figure 5 as the shift of the SPR under the thickest TiO_2_ cover is practically the same for all the samples.

The spectral shift of the SPR saturates when the electric field *E* generated by a nanoisland under probing electromagnetic wave is completely localized within the covering film and the glass substrate as shown in Figure 9 (inset c). For thinner TiO_2_ films, the tail of the SPR electric field penetrates through the covering layer, that is, the electric field is partly localized in the air (see Figure 9, inset b). In other words, the effective dielectric permittivity of the nanoisland surrounding is less for thinner covers than for thicker covers. This results in weaker dielectric loading of the SPR and corresponds to its unsaturated spectral shift, which tends to saturate with the TiO_2_ film thickness increase. Thus, the saturated SPR shift indicates that the thickness of the cover exceeds the length of the SPR electric field penetration into the cover (Figure 9, inset c).

As measured with absorption spectroscopy, the spectral shift of the SPR in TiO_2_-covered MIF saturates at about 40- to 50-nm cover thickness. We can suppose that the SPR electric field intensity decays in TiO_2_ film at about the same length. Unfortunately, comparing the dependences of the SPR spectral shift in Figure 5, one can hardly conclude whether there is a difference in the SPR decay length for differently prepared MIFs. The measured Raman scattering signal *I*_Raman_ should decay much faster. If the glass surface is covered with silver nanospheres, $I_{\text{Raman}}\sim {\left(\frac{r}{r+d}\right)}^{12}$[28] for separate molecules and $I_{\text{Raman}}\sim {\left(\frac{r}{r+d}\right)}^{10}$[29] for a monolayer of an analyte, where *r* is the radius of silver microsphere and *d* is the distance from the microsphere to the analyte. Definitely, one can see a very fast Raman signal decay in Figure 6 where the decrease in the signal relative to the uncovered MIF is presented. The decay is due to the spacer thickness influence and due to the absence of CHEM input (if any in the present case). At the same time, the spacer protects the MIF providing its longer time stability.

The increase in MIF density, that is, in size and in surface concentration of nanoislands, should result in a higher SERS signal (Figure 6). This is because of (a) the increase of the cross section of the nanoisland-analyte interaction due to a geometrical factor, that is, the increase of the effective area of the MIF, and (b) the surface concentration of ‘hot spots’ which are supposed to be the main origin of extremely high SERS signals [30,31]. This can be easily seen in Figure 6a where a denser film provides higher *I*_Raman_. At the same time, the increase in the size of nanoislands, indicated by the redshift of the SPR (Figure 4), and their coagulation definitely result in the slowing of the spatial decay of the SPR electric field with the spacer thickness. Figures 7 and 8, where one can see that the Raman signal decay with the spacer thickness is slower for the denser film, clearly illustrate this. This phenomenon can be very roughly explained through the increase in the effective size of nanoislands *d*, but its detailed description will definitely require accounting for peculiarities related to the redistribution of local SPR fields in the partly aggregated MIF [32]. It is worth to note that thicker TiO_2_ films, corresponding to full decay of the local electric field within the spacer, exclude SERS-related applications of the MIFs. However, they can be effectively used in applications which do not require the use of the tail of the electric field outside the film. Examples of such applications include tuning of optical absorption spectra, enhancement of resonant luminescence of emitters embedded into the film, and tuning the wavelength range of optical nonlinearity.

## Conclusions

The performed studies demonstrate that silver nanoisland films formed using out-diffusion of silver from glass substrates during thermal processing in hydrogen atmosphere can be effectively used in SERS measurements. The enhancement of the Raman signal increases with the density of the nanoisland film. The surface profile of dielectrics deposited upon the MIF using the ALD technique replicates the profile of the initial MIF, and the smoothing of the dielectric surface profile with the deposited thickness is rather slow except for the smallest gaps between the nanoislands. The deposition of a titanium dioxide film results in a redshift of the SPR wavelength relative to the SPR wavelength of the initial film. This shift is up to hundred nanometers allowing the tuning of the central wavelength of the SPR. The shift saturates at a titania film thickness of 40 to 50 nm. SERS experiments performed with a R6G probe show that the SPR field spatial decay is less for denser MIFs, that is, for these MIFs, the titania spacer can be thicker. Finally, catalytically active ALD-TiO_2_ films can be effectively used to protect very fragile silver nanoislands from sulfidation, oxidation, pollutions, etc., allowing the maintenance of the SERS properties of the MIF. Additionally, this allows the fine-tuning of the SPR position and, respectively, conditions for surface-enhanced resonant Raman scattering (SERRS).

## Abbreviations

AFM: atomic force microscopy; ALD: atomic layer deposition; CHEM: chemical enhancement; MIF: metal island film; SERS: surface-enhanced Raman scattering; SPR: surface plasmon resonance.

## Competing interests

The authors declare that they have no competing interests.

## Authors’ contributions

SC prepared the nanoisland film samples, measured the absorption spectra, and processed the resonance shift calculations. AM deposited the TiO_2_ on the samples and measured the Raman spectra. AD performed the AFM studies of the samples. AAL and SH supervised the whole work. All authors read and approved the final manuscript.
